# Hybrid Odontogenic Tumor of Calcifying Odontogenic Cyst and Ameloblastic Fibroma: a Case Report and Review of Literature

**DOI:** 10.30476/DENTJODS.2019.77806.

**Published:** 2020-06

**Authors:** Nazanin Mahdavi, Neda Kardooni Khoozestani, Mahboube Hasheminasab, Nika Soltani

**Affiliations:** 1 Dept. of Oral and Maxillofacial Pathology, School of Dentistry, Tehran University of Medical Sciences, Tehran, Iran; 2 Craniomaxillofacial Research Center, Dept. of Oral and Maxillofacial Surgery, School of Dentistry, Tehran University of Medical Sciences, Tehran, Iran; 3 Postgraduate student, Dept. of Endodontics, Faculty of Dentistry, Tehran Medical Science Islamic Azad University, Tehran, Iran

**Keywords:** Odontogenic tumor, Calcifying odontogenic cyst, Ameloblastic fibroma

## Abstract

Calcifying odontogenic cyst is an uncommon odontogenic lesion that represents less than 2% of all odontogenic cysts and tumors. It usually occurs in incisor and canine areas during the second to fourth decades of life. It can be associated with other lesions like odontoma, ameloblastic fibroma, ameloblastoma, adenomatoid odontogenic tumors, odontoameloblastoma, and odontogenic myxoma. Ameloblastic fibroma is a truly mixed tumor usually diagnosed within the posterior mandible during the first two decades of life. In the present article, a hybrid odontogenic tumor composed of calcifying odontogenic cyst and ameloblastic fibroma in a 14-year-old white Persian female is described.

## Introduction

Calcifying odontogenic cyst (COC) was first identified as a distinct pathologic entity by Gorlin *et al*. [ 1 ] in 1962. COC is an uncommon lesion, representing less than 2% of all odontogenic cysts and reveals a variety of clinical behaviors as well as histologic features that range from a cystic lesion to a solid tumor [ 2 ]. According to the World Health Organization (WHO) classification in 2005, this lesion is reclassified as a calcifying cystic odontogenic tumor (CCOT) [ 3 ]. COC is usually diagnosed during the second to fourth decades of life in the incisor and canine areas [ 4 ]. Radiographically, it usually appears as a unilocular and less commonly multilocular radiolucent lesion with well-defined borders [ 1 , 5 ]. Microscopically, the lesion reveals ameloblast-like epithelial cells with columnar basal cells. The most characteristic feature of COC is the presence of ghost cells within the epithelium that can undergo calcification [ 6 ]. The epithelial lining of COC can induce dentin formation in the adjacent connective tissue and association of COC with odontoma is relatively common. COC has also been reported in association with different odontogenic tumors [ 7 ]. Ameloblastic fibroma (AF) is a rare odontogenic tumor arising from both mesenchymal and ectodermal components of the tooth forming tissue [ 1 ]. AF is usually found in the posterior area of the mandible during the first two decades [ 4 ]. In the present article, a hybrid odontogenic tumor composed of COC and AF in a 14-year-old white Persian female is described.

## Case Report

**Figure 1:**
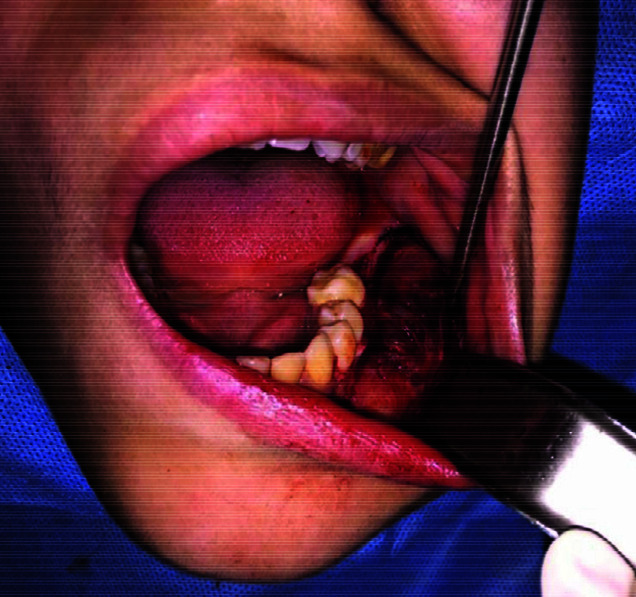
The expansile lesion in the left posterior area of the mandible

A-14-year old white Persian female was referred to the oral and maxillofacial surgery department of Tehran University of Medical Sciences, Tehran, Iran, with the chief complaint of purulent discharge from the left posterior mandible. On physical examinations, an expansile lesion in the left side of the mandible was found (Figure 1). General physical status was normal and there was no problem in the past medical history. The patient reported no neurosensory disturbances of the lower lip and chin. The left first molar had grade 3 of mobility. 

**Figure 2:**
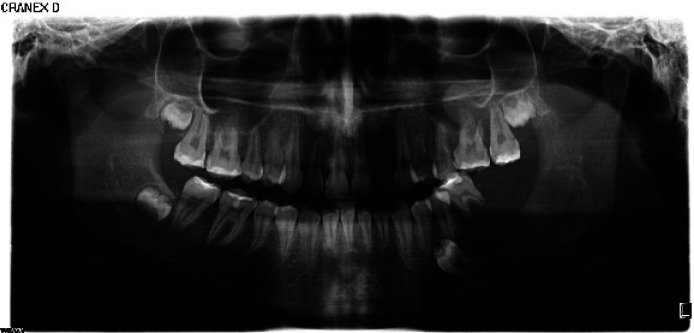
The panoramic radiograph showing a well-defined radiolucent lesion in the left mandibular body

**Figure 3:**
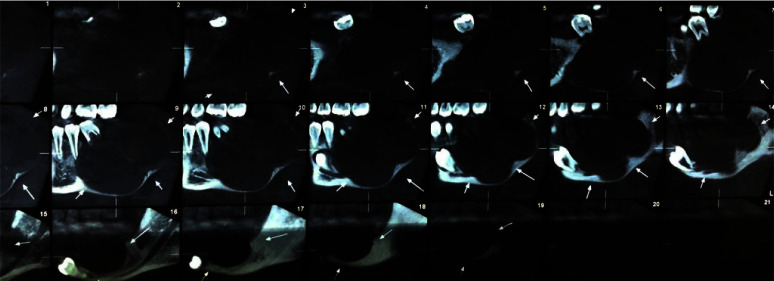
Sagittal view of the lytic lesion in CBCT

**Figure 4:**
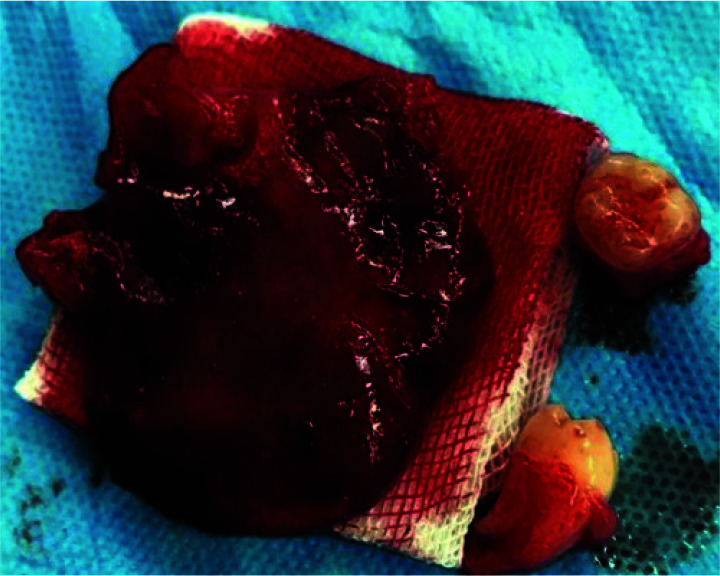
The lesion with the first and second molar teeth

**Figure 5:**
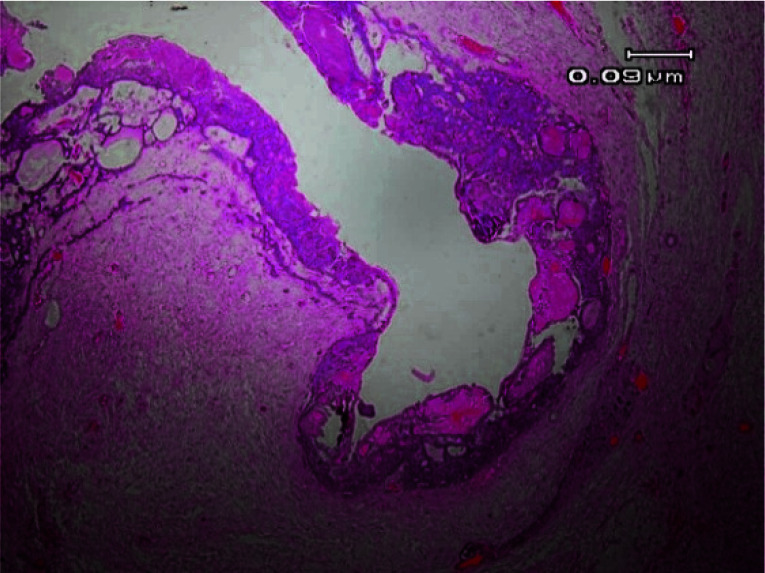
H&E stained sections; cyst walls lined by odontogenic epithelium demonstrating columnar cells with hyperchromatic nuclei in the basal cell layer and sheets of the ghost cells. (×40)

**Figure 6:**
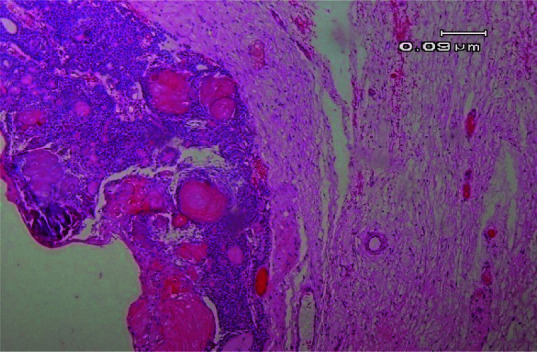
Sheets of Gorlin cells (×100)

**Figure 7:**
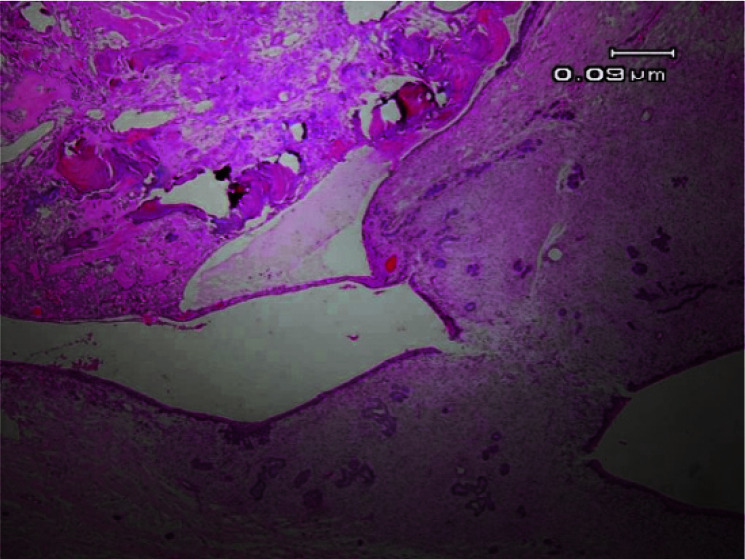
Foci of cell-rich mesenchymal tissue resembling primitive dental papilla, composed of plump stellate cells within a loose matrix with cords of proliferating odontogenic epithelium in the cyst wall (40×)

**Figure 8:**
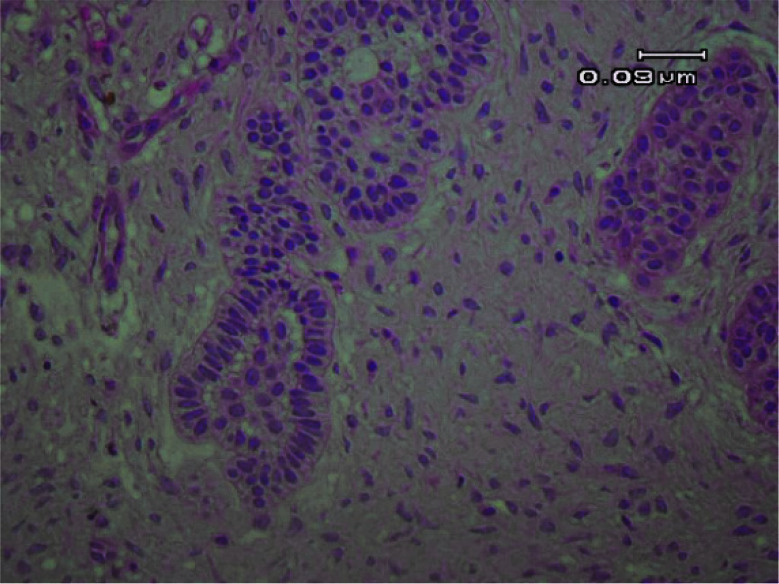
The epithelial cords composed of two layers of cuboidal cells that show juxta-epithelial hyalinization in some parts. (400×)

**Figure 9:**
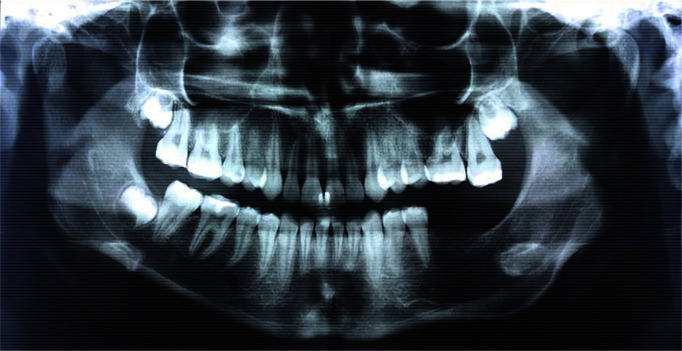
Panoramic view 20 months after the excision of the lesion

Radiographically, a unilocular radiolucent lesion was evident with well-defined borders, extending from the mesial aspect of the left first premolar tooth to the mid ramus area. The lesion pushed the mandibular canal downward and caused root resorption of the second premolar and the first molar teeth. Mesial and downward displacement of the second molar tooth was also evident. (Figure 2 and 3). Based on the clinical and radiographic presentations, odontogenic keratocyst (OKC) was considered as the main differential diagnosis and the lesion was excised in conjunction with the first and second molar teeth (Figure 4). Grossly the specimen consisted of a cystic lesion with elastic consistency, measuring 5×4×2.5 cm. Maximum thickness of the cyst wall was 0.6 cm and the lumen contained a viscous pasty material. Microscopic examinations demonstrated a cystic lesion with a thick fibrous wall, lined by odontogenic epithelium composed of cuboidal to columnar basal cells, and loosely arranged, stellate reticulum-like cells on the surface. Presence of numerous eosinophilic ghost cells within the epithelium was notable (Figure 5 and 6). In the cyst wall, foci of cell-rich mesenchymal tissue resembling primitive dental papilla, composed of plump stellate cells within a loose matrix admixed with cords of proliferative odontogenic epithelium were seen. The epithelial cords were composed of two layers of cuboidal cells that showed juxta-epithelial hyalinization in some parts (Figure 7 and 8). Based on the diverse histopathologic features of the lesion, the diagnosis of hybrid odontogenic tumor composed of COC and AF was established. After the surgical excision of the lesion, the patient reported a partial loss of sensitivity in the lower lip which improved in less than a month. The patient has been recalled every three months for 20 months, and no recurrence has been detected (Figure 9). Informed consent was obtained from the patient for publishing her clinical photography and radiography.

## Discussion

COC is an uncommon odontogenic lesion which was first identified as a distinct pathologic entity by Gorlin *et al*. [ 1 ] in 1962. Despite the fact that AF is more common during the first two decades, most cases of COC are diagnosed during the second to fourth decades of life. In this case, the hybrid tumor of COC and AF occurred in a teenage girl who is in the common age for AF [ 4 ]. COC has been reported in association with different odontogenic tumors including odontoma, the most common ameloblastoma, adenomatoid odontogenic tumors, odontoameloblastoma, ameloblastic fibroma, and odontogenic myxoma [ 5 , 7 ]. So far, there have been seven articles of hybrid COC and AF in the English literature, which are listed in Table 1.

**Table 1:** Reported cases of COC with AF

Author	Year	Number of the cases	Age	Sex	Location	Association with an unerupted tooth	Sign and symptoms
Shear M. [12]	1976	1	-	-	-	-	-
Farman *et al*. [13]	1978	1	42	Female	Mandible, anterior, crossing the midline	No	Painless swelling
Prætorius *et al*. [5]	1981	1	17	Male	Mandible ,molar and premolar area	Yes	Swelling
Yoon *et al*. [14]	2004	1	22	Female	Maxilla, molar area	No	Tooth mobility and displacement, swelling, discharge
Lin *et al*. [10]	2004	3	6	Female	Mandible ,molar area	No	Painless swelling
			13	Male	Maxilla, molar area	Yes	Swelling with dull pain
			22	Male	Mandible, molar area	Yes	Not mentioned
Phillips *et al*. [15]	2010	1	7	Male	Mandible, anterior, left	No	No sign and symptoms
Neuman *et al*. [16]	2015	1	10	Male	Mandible, angle and ramus	No	Pain and swelling

The exact mechanism that creates these combinations is not well understood. A number of possible mechanisms have been suggested including a collision of two separate lesions, a transformation of one lesion into another and an induction of one lesion by the other one [ 8 ]. Altini and Farman [ 9 ] suggested that the development of COC results from transformative changes within a pre-existing odontogenic tumor. The epithelial lining of COC has the ability to inactivate the adjacent connective tissue and induce dentin formation [ 10 ]. It has been suggested that the development of another odontogenic tumor in association with COC is induced by the odontogenic epithelial islands within the connective tissue wall of COC [ 5 ]. In the present case, AF is developed in the connective tissue wall of COC and is located subjacent to the epithelium in most parts, which supports the possibility of induction of AF by the epithelium of COC. COC is usually treated by simple excision with a good prognosis and when it is associated with other odontogenic tumors, the treatment is based on the accompanied tumor [ 11 ]. In this case, the lesion was excised more aggressively because of the presence of AF. Prognosis of this case will be probably similar to that of AF, although long term follow-up is needed to determine the clinical significance of the presence of AF in association with COC. In this case, the lesion was excised completely, and the patient has been followed for 20 months without any signs of recurrence.

## Conclusion

COC is a rare odontogenic cyst, which can be accompanied by other cysts and tumors like AF. In this case, a hybrid tumor of COC and AF was diagnosed in a 14-year-old teenage girl; presenting an uncommon age for COC considering the literature. The treatment plan was based on AF treatment modality, subsequently; the lesion was excised completely with regular follow-ups. 
